# Development of IgY-Based Indirect Competitive ELISA for the Detection of Fluoroquinolone Residues in Chicken and Pork Samples

**DOI:** 10.3390/antibiotics11111512

**Published:** 2022-10-29

**Authors:** Sumed Yadoung, Ryoichi Ishimatsu, Zhen-Lin Xu, Korawan Sringarm, Supansa Pata, Marninphan Thongkham, Somporn Chantara, Mookda Pattarawarapan, Surat Hongsibsong

**Affiliations:** 1 Environmental Science Program, Faculty of Science, Chiang Mai University, Chiang Mai 50200, Thailand; 2 Department of Applied Chemistry, Graduate School of Engineering, Kyushu University, Motooka, Nishiku, Fukuoka 819-0395, Japan; 3 Guangdong Provincial Key Laboratory of Food Quality and Safety, South China Agricultural University, Guangzhou 510642, China; 4 Department of Animal and Aquatic Sciences, Faculty of Agriculture, Chiang Mai University, Chiang Mai 50200, Thailand; 5 Division of Clinical Immunology, Department of Medical Technology, Faculty of Associated Medical Sciences, Chiang Mai University, Chiang Mai 50200, Thailand; 6 Department of Chemistry, Faculty of Science, Chiang Mai University, Chiang Mai 50200, Thailand; 7 School of Health Sciences Research, Research Institute for Health Sciences, Chiang Mai University, Chiang Mai 50200, Thailand

**Keywords:** antibiotics residue, fluoroquinolones, ciprofloxacin, norfloxacin, enrofloxacin, indirect competitive enzyme-linked immunosorbent assay (icELISA), IgY antibody

## Abstract

Fluoroquinolones (FQs) are among the antibiotics whose widespread use in farm-raised animals results in potentially harmful residues in the end products. Additionally, most Thai farmers use antibiotics. Amoxicillin and enrofloxacin were commonly used by pig farms, and hens were given enrofloxacin to prevent immunization side effects. Moreover, antibiotic overuse has harmed food safety in the long term, and the use of low-dose antibiotics causes bacterial resistance. Herein, an indirect competitive enzyme-linked immunosorbent assay (icELISA) was used to make a fast, easy, sensitive, and cost-effective method for monitoring FQs residues. After immunizing hens with mixed multi-hapten ciprofloxacin-bovine serum albumin (CPFX-BSA) with norfloxacin-bovine serum albumin (NFX-BSA), the IgY antibody purified from egg yolk was used for the detection of FQs residues in chicken and pork samples. The efficiency of the IgY antibody showed excellent sensitivity, with 50% inhibitory concentration (IC_50_) of enrofloxacin at 0.05 µg/mL, far below the MRLs defined by the EU for muscle samples, which was not to exceed 100 µg/kg. The recovery range for chicken muscle samples spiked with ENFX concentrations of 1.00–0.01 µg/mL was 86.65–112.71%, similar to pork samples, which were 84.24–117.22.2%. This method has a lot of potential for analyzing fluoroquinolones in complex samples quickly, easily, and at a low cost on-site. The IgY-based ic ELISA was developed to detect ciprofloxacin (CPFX), norfloxacin (NFX), and enrofloxacin (ENFX) residues; it confirms that IgY could be a promising choice for the detection of antibiotic residues in food samples.

## 1. Introduction

The fluoroquinolones are one of the antibiotics that act as broad-spectrum agents that act through the inhibition of an essential bacterial enzyme, DNA gyrase, abolishing its activity by interfering with the DNA rejoining reaction [1].

Ciprofloxacin (CPFX) is a fluoroquinolone antibiotic commonly prescribed to treat infections caused by Gram (+) and Gram (−) microorganisms, particularly infections of soft, respiratory, and bacterial tissues, in addition to sexually transmitted diseases and sinusitis [2].

Norfloxacin (NFX) is an often-reported fluoroquinolone antibiotic used to treat urinary bacterial infections in veterinary patients. NFX is widely used in livestock for preventing disease and promoting growth. However, the long-term accumulation of NFX in the human body as a consequence of consuming food containing NFX residues might readily result in phototoxic side effects [3,4,5].

Enrofloxacin (ENFX) is the first fluoroquinolone designed specifically for veterinary usage. This belongs to the second generation of fluorinated quinolone antibiotics. ENFX is used to treat systemic infections, including infections of the urinary tract, respiratory system, gastrointestinal tract, and skin. Because of a very broad spectrum of activities against both Gram-negative and Gram-positive bacteria and fewer side effects, ENFX has also been widely used for the treatment of some infectious diseases in pets and livestock [6].

In the past, several quinolones have been authorized for the treatment of cattle, poultry, fish, and other animals in various nations. In China, it is estimated that approximately 500 tons of quinolones are used annually in veterinary medicine. As is as the case in Turkey, pork and chicken meat was sampled, and more than 50% of the total samples were found to contain quinolone residue. Due to a large volume of quinolone antibiotics usage, there is a high possibility that antibiotic residues will be found in meat products. If sufficient checks and balances are not implemented, it is possible for poultry and pork tissues to be contaminated with hazardous quantities of antibiotic residues [7,8].

The widespread usage of multiple quinolones in animals grown on farms can result in potentially hazardous residues in the final products. The extensive abuse of antibiotics has caused severe food safety problems. Meanwhile, some research indicates that low-level doses of antibiotic exposure for long periods could result in bacterial resistance. The concern for antibiotic residue in animal products and maximum residue limits (MRLs) for several antibiotics has been established in many countries to protect consumers. CPFX, NFX, and ENFX residues in livestock products may cause serious public health problems by persisting in an animal’s body and may result in the development of drug-resistant bacterial strains or allergies [9].

Over 80% of animal producers in Thailand utilize antibiotics, which have applications in animal growth promotion, disease prevention, and treatment. Pig farmers used amoxicillin and enrofloxacin as the most common antibiotics to treat diarrhea or lung infections in pigs. Enrofloxacin is administered to all new laying hens by laying-hen farmers in order to prevent vaccination-related side effects. Therefore, the consumption of animal products containing quinolone residues may facilitate the proliferation of resistant microbes. In addition, quinolone active metabolites are harmful to human health if their residual levels exceed tolerance thresholds [10,11,12].

The standard method for detecting antibiotic residue is chromatographic-based methods such as high-performance liquid chromatography (HPLC), liquid chromatography/mass spectrometry (LC–MS), and LC–MS/MS. However, chromatography has many limitations such as being time-consuming and the high cost of the instruments. Moreover, the sample pretreatments are complicated and difficult for field operations. Therefore, alternative methods for the rapid screening of large numbers of samples of residues are needed. Among the alternative methods of measurement, immunoassays have been widely used because of high sensitivity and rapidity, lower sample dosage, and operation simplicity [13].

Chicken immunoglobulin Y (IgY) is a highly conserved homolog of human immunoglobulin G (IgG) that has shown benefits and a favorable safety profile, primarily in animal models of human infectious diseases. IgY is fast-acting, easy to produce, and low cost. IgY antibodies can be readily generated in large quantities with minimal environmental harm or infrastructure investment by using egg-laying hens [14].

**Scheme 1:**
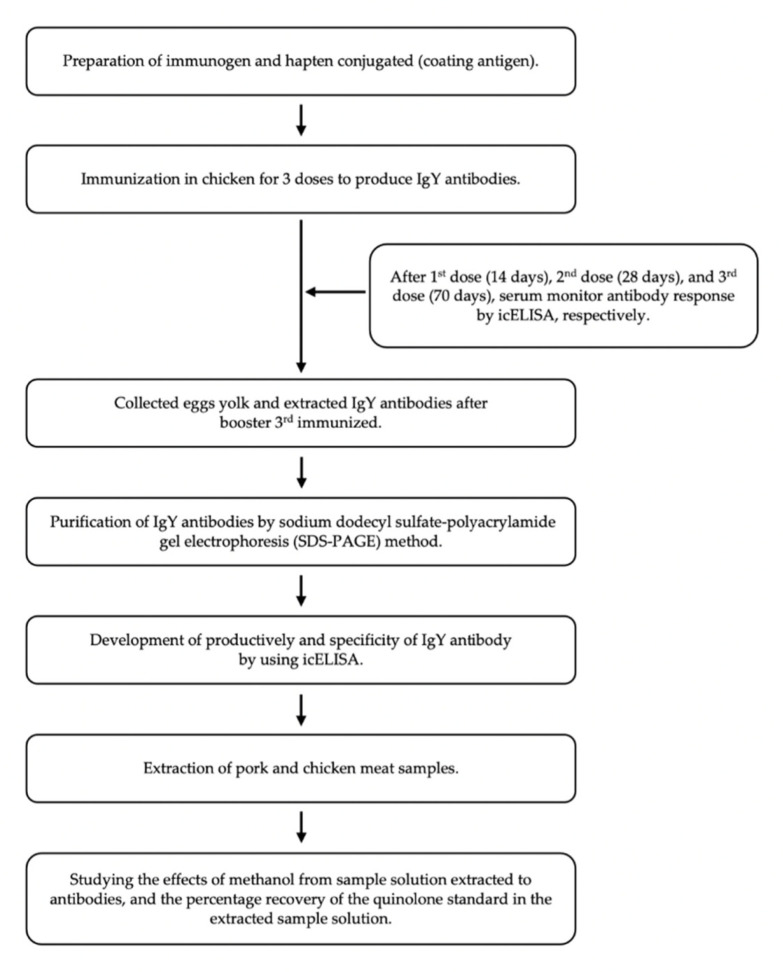
The process of running the current study.

Herein, the current study developed an immunoassay by using the IgY antibody from immunized chicken with immunogen-stimulated hen egg yolks. In addition, studied the productivity of IgY antibody specific to fluoroquinolones, i.e., ciprofloxacin, norfloxacin, and enrofloxacin. The indirectly competitive ELISA was developed for detecting quinolone antibiotics and for detecting residue in chicken and pork meat samples. The process of this study is shown in Scheme 1.

## 2. Results and Discussion

### 2.1. Immunogen and Coating Antigen Characterization

**Figure 1:**
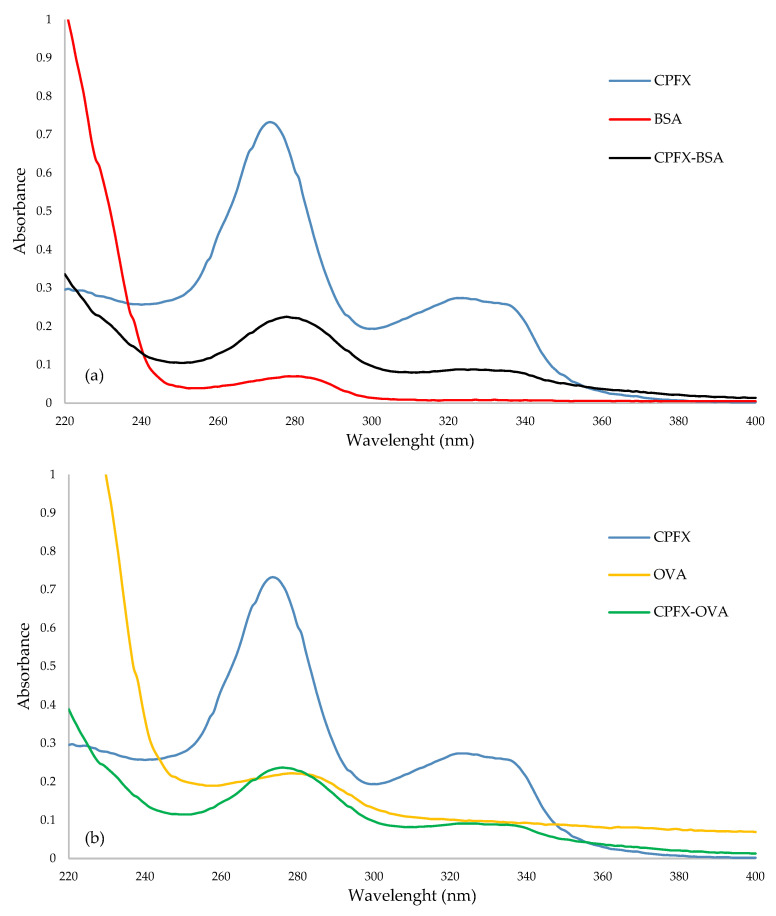

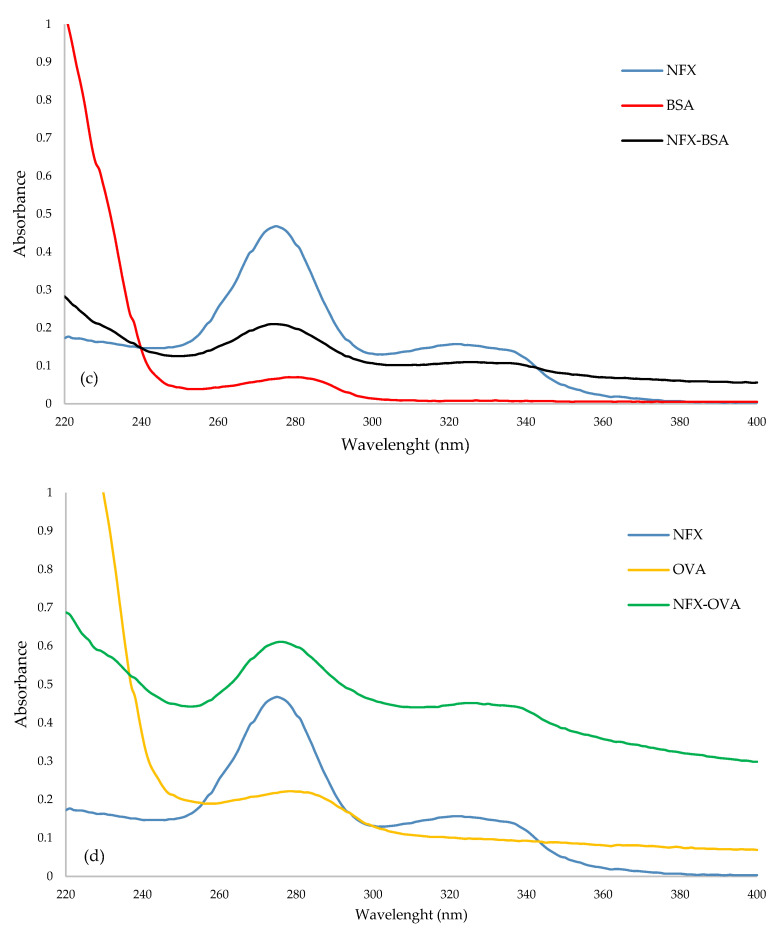
The UV–visible absorption spectra of immunogen and the coating antigen was characterized by spectrophotometry: (**a**) spectrum of CPFX, BSA, and immunogen conjugates (CPFX-BSA); (**b**) spectrum of CPFX, OVA, and the coating antigen (CPFX-OVA); (**c**) spectrum of NFX, BSA, and immunogen conjugates (NFX-BSA); (**d**) spectrum of NFX, OVA, and the coating antigen (NFX-OVA).

In this study, ciprofloxacin (CPFX) and norfloxacin (NFX) are small molecules with a molecular weight of 331.346 and 319.331 g/mol. To prepare the immunogen and the coating antigen, this molecule was conjugated to protein carrier molecules. BSA and OVA were used as carriers. CPFX and NFX were conjugated with BSA and OVA to produce the immunogen and coating antigens, respectively. Coupling efficiencies were evaluated by UV spectroscopy, as shown in Figure 1a,b, which shows the absorption peaks of CPFX, BSA, and OVA at 273, 278, and 278 nm, respectively. Conversely, CPFX-BSA appeared as a broader peak than BSA at 278 nm and exhibited the same linear trend as CPFX from 220 to 450 nm due to the influence of CPFX groups linked to BSA, which is also the same result as CPFX-OVA. Figure 1c,d shows the absorption peaks of NFX at 275 nm. Furthermore, BSA and OVA absorption peaks are the same as in Figure 1a,b. Alternatively, the broad peaks of NFX-BSA and NFX-OVA are wider than BSA and OVA at 278 and 278 nm, respectively. The absorption curves of CPFX-BSA and CPFX-OVA obviously differed from BSA and OVA, respectively, and appeared to have the characteristic absorption of CPFX groups, and the results of NFX-BSA and NFX-OVA were similar. Therefore, the immunogens CPFX-BSA, CPFX-OVA, NFX-BSA, and NFX-OVA were prepared successfully, and cationized proteins modified with diamines to increase their pI are known to generate an increased immune response compared to their native forms [15,16].

Moreover, the quantity of haptens covalently bound to the surface of a carrier molecule is crucial for vaccine effectiveness. The hapten density of all of the samples (ciprofloxacin and norfloxacin conjugated) were purified by dialysis. By trinitrobenzenesulfonic acid (TNBS) assay [17], the hapten density of CPFX-BSA, CPFX-OVA, NFX-BSA, and NFX-OVA conjugated were 0.0030, 0.0021, 0.0107, and 0.0226, respectively, as shown in Table 1.

### 2.2. Production of Antibody to Fluoroquinolone Antibiotics

#### 2.2.1. The Immunoglobulin G Response in Chicken Hen Serum after Immunization

Because antibiotics in the quinolone group have a molecular weight more than 300 and have a carboxylic group for coupling with the protein carrier. Thus, several previous works usually took advantage of a 3-carboxyl group on the main nucleus [18]. The method was not suitable due to the fact that the main construction of quinolones was masked. Antibodies to one medicine could be acquired through the way described above instead of broad-specificity antibodies to quinolones. The CR might be appropriate on account of the similar conformation.

**Figure 2:**
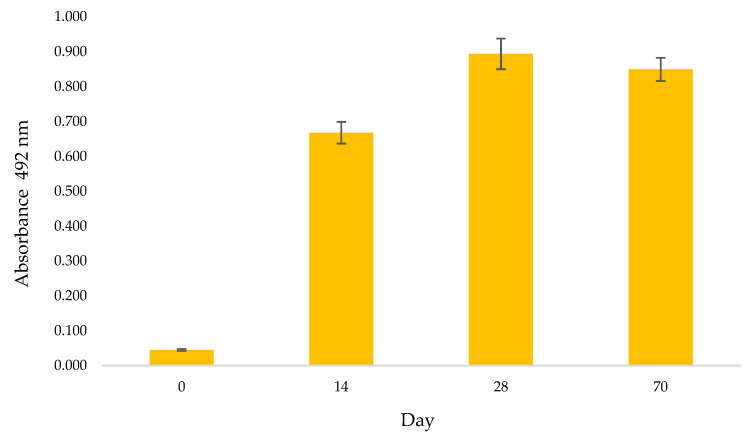
Time-dependent variations in the serum activity of a particular antibody during the experiment. Chickens’ antibody activities in 1:1000 dilution was measured by ELISA and shown as absorbance 492 nm, and error bars indicate the standard deviation (n = 2).

Regarding the immunogen in the different immunization steps, immunogen was used. After each immunization, the immune response of the chicken hen was checked by an indirect ELISA using serum. All three immunized hens reacted clearly toward the administered immunogen. After the immunization of the chicken was completed, the responsibility of the IgG antibody from chicken serum was assessed for time-interval immunizations at days 0, 14, 28, and 70, respectively. The activity is shown in Figure 2, obtained by measuring 1:1000 dilution serum chicken antibody by the ELISA method and absorption at 492 nm. Figure 2 shows that the activity increases significantly from day 0 to day 14 and then again from day 14 to day 28. The activity value slightly decreasing 42 days later demonstrates that hens’ immune systems react well to immunogens.

#### 2.2.2. Preparation of Immunoglobulin Y from Egg Yolk and the Characterization of Sensitivity and Specificity

**Figure 3:**
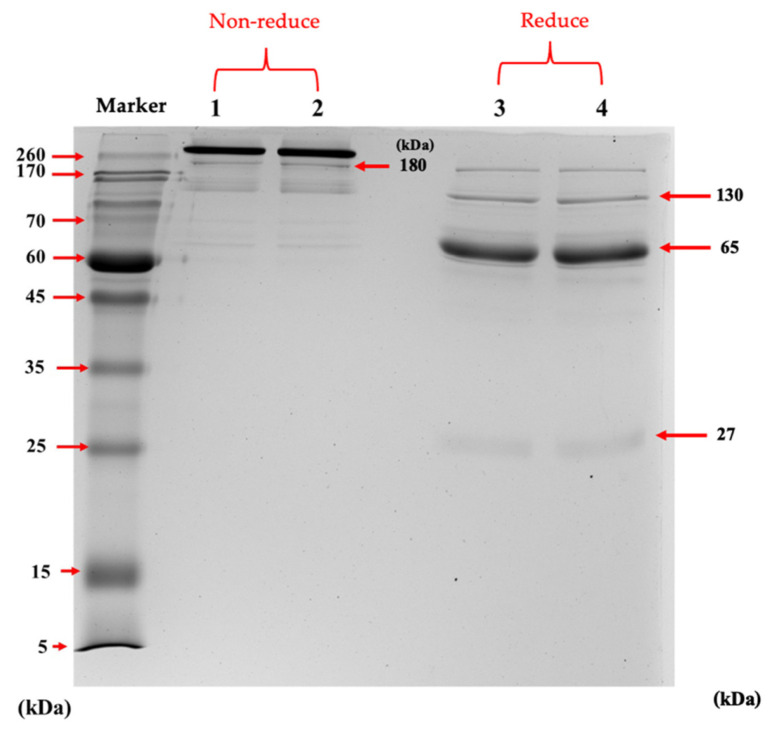
SDS–PAGE visualized intact IgY released from the antibody extracted from egg yolk. The HC heavy chain with 27, 65, and 130 kDa under reducing conditions and 130 kDa under non-reducing conditions appeared on the SDS–PAGE using 10% resolving SDS–PAGE gel.

The eggs were collected after the third immunization, and yolks were purified by using PEG8000. After purification, the molecular weight and purity of the IgY antibody were checked by sodium dodecyl sulfate–polyacrylamide gel electrophoresis (SDS–PAGE). The result was utilized to evaluate the water-soluble fractions produced following IgY extraction. According to the protein ladder, the estimated molecular mass for the heavy chains of egg yolk antibody IgY was 27, 65, and 130 kDa under reducing conditions and 130 kDa under non-reducing conditions. The molecular weight patterns corresponded with this value (Figure 3). Using SDS–PAGE, we demonstrated that the pure fraction in our chromatography results corresponded to the expected sizes of IgY heavy chains. Consequently, the 27, 65, and 130 kDa heavy chain was validated by SDS–PAGE.

#### 2.2.3. The Characteristic of IgY Antibody by Using Indirect Competitive ELISA

In order to avoid the suffering that animals endure during the synthesis of particular antibodies, IgY technology has been proposed as a substitute method for obtaining polyclonal antibodies in mammals. IgY has a long history of use in many different applications. The goal of this study was to create an IgY-based icELISA for the detection of fluoroquinolone antibiotic residues in chicken and pork samples, specifically ciprofloxacin, norfloxacin, and enrofloxacin.

Throughout the course of the immunization of hens, chicken egg yolk is a plentiful and consistent source of antibodies, and the antibody titer may still be strong even after 24 months. The principles of replacement, reduction, and refinement are also adhered to by IgY, which is also noninvasive.

**Figure 4:**
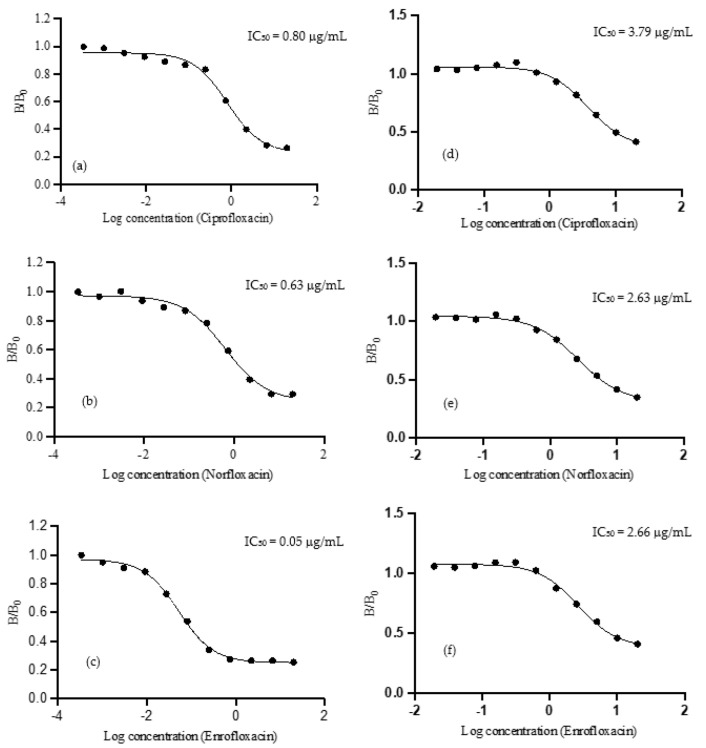
The inhibition curve of fluoroquinolone antibiotics using mixed CPFX–BSA and NFX–BSA activation of the antibody, (**a**–**c**) using CPFX–OVA as the coating antigen; (**d**–**f**) using NFX– OVA as the coating antigen.

The ELISA was developed, and several assay parameters were optimized by checkerboard analysis. The concentration of the coating antigen was found to have the highest impact on the sensitivity of the assay. The optimal coating concentration was found to be 20 µg/mL. The optimal concentration of the purified IgY antibody was 1/4000. The optimal secondary antibody concentration (anti-chicken IgY-HRP) was 1/10,000. The concentrations of CPFX, NFX, and ENFX that give half of the maximum signal intensity (IC_50_) were determined. The standard inhibition curve for antibiotics generated using this ELISA system with optimized conditions is shown in Figure 4. Eleven concentrations of antibiotics were applied to the ELISA system by coating CPFX-OVA, and the consequence is the mean values of IC_50_ for CPFX, NFX, and ENFX were 0.80, 0.63, and 0.05 μg/mL, respectively. Moreover, the mean values of IC_50_ by coating NFX-OVA was shown as well, for CPFX, NFX, and ENFX. These values were 3.79, 2.63, and 2.66 μg/mL, respectively.

**Figure 5:**
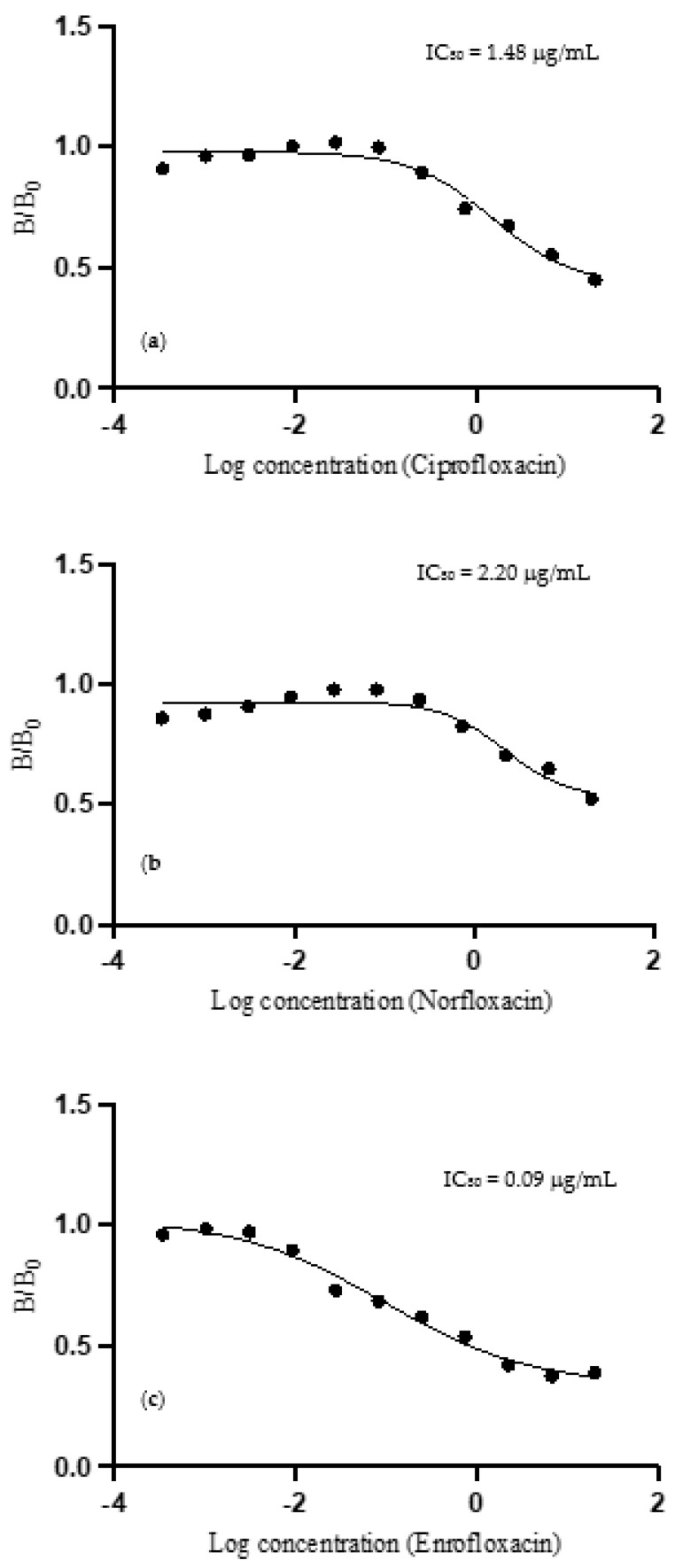
The inhibition curve of fluoroquinolone antibiotics by using the CPFX–BSA activation of the antibody and CPFX–OVA as the coating antigen; (**a**) ciprofloxacin; (**b**) norfloxacin; (**c**) enrofloxacin.

According to the results of different immunogen-stimulated antibodies triggered by CFPX conjugated from a graph in Figure 5, the IC_50_ values are higher than the other antibodies shown in Figure 4, which are against activated antibodies mixed with NFX and CPFX conjugated. Therefore, the result of the study showed that antibodies derived from multiple hapten activations were more effective and specific than those activated by a single hapten.

#### 2.2.4. Antibody Specificity

The specificity of the antibody was evaluated by performing competitive assays against three fluoroquinolone compounds, and the IC_50_ values (μg/mL) obtained were utilized to calculate the percentage of cross-reactivity. The IC_50_ values obtained were used to calculate (Equation (1)) the percentage cross-reactivity of each compound to CPFX. The results summarized in Table 2 represent a mean of two separate experimental runs, each containing duplicates. Antibody specificity was determined by the indirect competitive ELISA in which CPFX competitors, two quinolone derivatives, ENFX and NFX, were present in the assay to compete for the binding of CPFX. The antibody demonstrated high affinity to its CPFX at 100%, although there was more than 125.87% affinity for the NFX and 1531.34% affinity for the ENFX as well.

The results of %CR of two coating antigens i.e., CPFX-OVA and NFX-OVA, are shown in Table 2. The coating antigen CPFX-OVA showed %CR with ENFX higher than using NFX-OVA. Thus, the CPFX-OVA and IgY from mixed antigens were selected and used for developing the heterologous ELISA and the application used for chicken and pork samples.

### 2.3. The Validation of Heterologous ELISA for Detecting Enrofloxacin in Chicken and Pork Meat Samples

#### 2.3.1. Methanol Effect on Antibody

**Figure 6:**
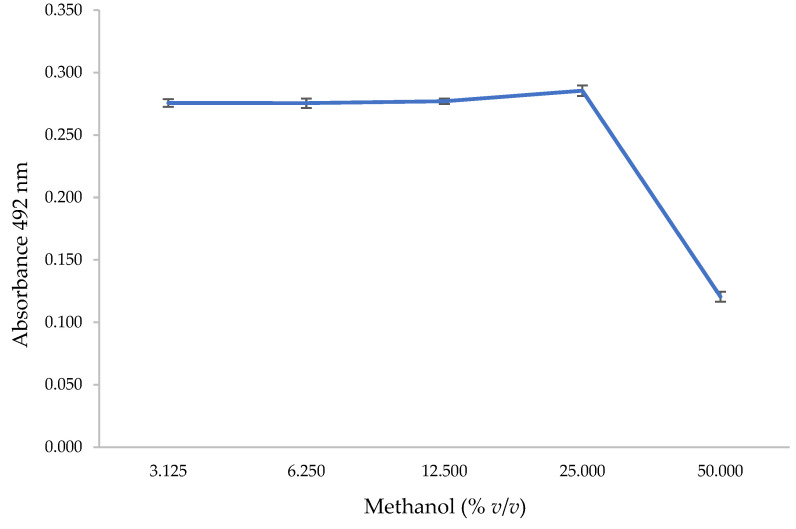
Effect of different percent methanol on the antibody activity of immunoassay (*n* = 3), methanol in 1% skim milk.

The CPFX-OVA was selected and used as the coating antigen, and the IgY antibody was tested for the methanol effect in an optimized icELISA system [19]. Methanol was used as the solvent for extracting the antibiotic from meat samples. Figure 6 shows the effects of methanol on antibody immunoassay activity. At concentrations of 3.125, 6.125, 12.50, and 25.00% methanol, the absorbance variations were negligible. In contrast to a methanol concentration of 50.00%, the absorbance was significantly decreased, representing a reduction rate of 42.23%. Therefore, the results demonstrated that a concentration of 50% methanol significantly affected the antibody’s activity. Considering the solution from pork and chicken extracted samples, the final concentration of methanol is 14%. Therefore, the results showed that the final concentration of methanol in the extraction solution had no effect on the antibody efficacy. Thus, it can be used to extract samples and analyze the antibiotic in samples.

#### 2.3.2. Recovery Study for ELISA in Matrix

Matrix interference is common in recovery studies for ELISA. In this investigation, matrix effects were significantly diminished by simple dilution. The meat samples (chicken and pork) spiked at three concentrations (1.00, 0.10, and 0.01 μg/mL) were analyzed, and the recovery result was tabulated (Table 3). The intra-assay recoveries for chicken in spiked concentrations of ENFX ranged from 111.81 to 112.71%, 96.69 to 103.62%, and 86.65 to 95.01%, respectively. Meanwhile, pork recoveries were spiked at concentrations ranging between 109.90 to 117.22%, 98.45 to 103.00%, and 84.24 to 87.90%, respectively. Thus, the ENFX has recovered adequately and reproducibly using this extraction method.

All of the tested quinolones showed a cross-reactivity of more than 120% cross-reactivity except enrofloxacin (1500%), indicating that the developed immunoassay had a high specificity for CPFX. The previous report also shows the results of developed immunoassays against sarafloxacin and ciprofloxacin. It had cross-reactivities ranging from 1 and to more than 100% with related fluoroquinolones. The high specificity observed is probably due to the small size of flumequine and the different structure in comparison with the other tested fluoroquinolones.

In this study, the heterologous ELISA for detecting ENFX in chicken and pork samples showed the good sensitivity with a low percent of cross-reactivity with another quinolone antibiotic. Heterologous ELISA is termed system indirect competitive ELISA to indicate the differences in hapten structure, linker attachment site, or bridge character, which usually results in the weaker recognition of antibodies to the coating antigen compared to the target compound, which can be the alternative format of the immunoassay for detecting small molecules [18,20]. The developed heterologous icELISA could use for detecting the sums of CPFX and ENFX at 30 µg/kg, which is the maximum residue limit set by EU regulations on edible animal tissues [21].

Table 4 summarized several immunoassay methods to determine enrofloxacin levels and provided comparative data for different methods and antibodies in the determination of enrofloxacin. Although the IC_50_ value of the enzyme-linked immunosorbent assay (ELISA) was larger than that of the other immunoassays, which were still considered acceptable, the immunoassay approach in this study yielded the same result, detecting values of IC_10_ to IC_90_ as 0.37 μg/mL and 7.29 ng/mL, respectively. Definitely, the LOD was set as IC_10_ at a concentration of 0.37 ug/mL as well. Because the value of maximum residue limits (MRL) established by the European Medicines Agency (EMA) was specified as not exceeding 100 μg/kg in muscle [22], the immunoassays were highly effective at detecting quinolone antibiotic residues, and some immunoassays were less effective than the usual chromatography approach. In addition, certain immunoassay methods exhibited a significant level of sensitivity, demonstrating that the immunoassay approach is comparable to the standard method.

## 3. Materials and Methods

### 3.1. Chemicals and Materials

Ciprofloxacin and norfloxacin standards were purchased from A.T. SCIENCE trading limited partnership (Sigma-Aldrich, St. Louis, MO, USA). Enrofloxacin was purchased from General Drugs House Co., Ltd., Lam Luk KA, Thailand. N-hydroxysuccinimide, NHS (Sigma-Aldrich, Darmstadt, Germany). 1-Ethyl-3-(3-dimethylaminopropyl) carbodiimide, EDC (Sigma-Aldrich, St. Louis, MO, USA). Dimethylformamide, DMF (Merck, Darmstadt, Germany). Bovine serum albumin, BSA (Sigma-Aldrich, Darmstadt, Germany). Ovalbumin, OVA (Sigma-Aldrich, Darmstadt, Germany). Dimethyl sulfoxide, DMSO (ACI Labscan, Bangkok, Thailand). Skim milk. Freund’s complete adjuvant, CFA (Sigma-Aldrich, Darmstadt, Germany). Freund’s incomplete adjuvant, FIA (Sigma-Aldrich, Darmstadt, Germany). Enzyme immunoassay-grade horseradish peroxidase-labelled goat anti-chicken immunoglobulin, HRP-IgY (Invitrogen, Rockford, IL, USA). O-phenylenediamine dihydrochloride, OPD (Sigma-Aldrich, St. Louis, MO, USA). Sulfuric acid, H_2_S0_4_ (J.T.Baker, Radnor, PA, USA). Sodium carbonate, Na_2_CO_3_ (Merck, Darmstadt, Germany)_._ Sodium Bicarbonate, NaHCO_3_ (Merck, Darmstadt, Germany). Polysorbate 20, Tween-20 (Loba Chemie, India). Sodium chloride, NaCl (ACI Labscan, Bangkok, Thailand). Potassium chloride, KCl (Sigma-Aldrich, Darmstadt, Germany). Disodium hydrogen phosphate Na_2_HPO_4_ (ACI Labscan, Bangkok, Thailand). Potassium dihydrogen phosphate KH_2_PO_4_ (Merck, Darmstadt, Germany). Citric acid, C_6_H_8_O_7_ (Merck, Darmstadt, Germany). 2,4,6-Trinitrobenzenesulfonic acid, TNBS (Sigma-Aldrich, St. Louis, MO, USA). L-glutamic acid C_5_H_9_NO_4_. Methanol (J.T.Baker, Radnor, PA, USA). Polyethylene Glycol 8000 PEG 8000 (Bio Basic, Markham, ON, Canada).

### 3.2. Solutions and Buffers

The buffer solution was prepared per liter of water. Phosphate buffer saline (PBS, pH 7.4) was prepared with NaCl (8.00 g), KCl (0.20 g), Na_2_HPO_4_ (1.15 g), and KH_2_PO_4_ (0.20 g). The coating buffer (pH 9.6) consisted of Na_2_CO_3_ (0.80 g), and NaHCO_3_ (1.50 g). The citrate–phosphate buffer (pH 4.0) contained C_6_H_8_O_7_ (21.00 g), and Na_2_HPO_4_ (14.20 g). The washing buffer contained 0.05% tween-20 in PBS (PBST). The ELISA substrate reagent freshly prepared was 10 mL of the 0.1 M citrate–phosphate buffer (pH 4.0) and 500 μL of the 0.5 mg mL^−1^ OPD solution containing 5 μL of 30% hydrogen peroxide. The ELISA stop reagent was 2 M sulfuric acid.

### 3.3. Preparation of Immunogen for Immunization and Coating Antigen for Immunoassay Development

Norfloxacin was conjugated to OVA and BSA by the carbodiimide active ester method [31]. The reaction contained norfloxacin 200 mg, N-hydroxysuccinimide (NHS) 100 mg, 1-Ethyl-3-(3-dimethylaminopropyl) carbodiimide (EDC) 10 mg, and dimethylformamide (DMF) 10 mL, stirred at room temperature for 30 min. Then, the solution was added dropwise to the OVA and BSA solutions (16 mg L^−1^ in 10 mL of PBS) in each tube and stirred at room temperature for 2 h. It was then dialyzed six times in PBS at 4 °C for three days.

Ciprofloxacin was conjugated to OVA, and BSA was prepared by the ester method [21]. Preparing solution 1, 2.3 mL of DMF was added and mixed with 38.6 mg of ciprofloxacin, 206.4 mg of EDC, and 57.6 mg of NHS. Then, the solution was incubated for 24 h at room temperature. Solution 2, 115 mg BSA, and OVA were mixed with 10 mL of pH 7.0, 0.01 M PBS in each tube. After that, both solutions were mixed by adding solution 1 into solution 2 slowly and shaking (120 rpm) for 3 h at room temperature. Finally, it was dialyzed six times in PBS at 4 °C for three days.

### 3.4. Antibody Production and Characterization

Two adult female chickens (*Gallus gallus domesticus*) were immunized subcutaneously; the first chicken was immunized with a mixture of 500 µg of CPFX-BSA and NFX-BSA, with a total volume of 500 μL (0.01 M PBS, pH 7.0); the other chicken was immunized with 500 µg of CPFX-BSA in a total volume of 500 μL (0.01 M PBS, pH 7.0). The first dose was emulsified with an equal volume of complete Freund’s adjuvant (CFA). Serum anti-CPFX and anti-NFX response was evaluated via indirect competitive ELISA (icELISA). The second and third dose was inoculated with 500 µg of CPFX-BSA and NFX-BSA in IFA twice at intervals of 7 days for subsequent boost immunizations. The level of IgG polyclonal antibody in serum measured after the first immunization at 14 days, 28 days (second dose), and 70 days (third dose) were monitored before collecting antibodies from egg yolks [32,33].

IgY antibodies were isolated from chicken egg yolks [34]. Egg yolks after three doses were combined after being dissolved in 10 mL of PBS (pH 7.4), and PEG 8000 was added, which was required to reach the desired final concentration of 3.5%, then vortexed until it was dissolved. After that, the mixture was rolled on a rolling mixer for a period of 20 min. The precipitate was removed after the mixtures were centrifuged at 13,000× *g* for 20 min at 4 °C. The supernatant was infused with PEG 8000 at a final concentration of 8.5%. The samples were mixed for 20 min on a rolling mixer. The mixtures were once more centrifuged for 20 min at 4 °C at 13,000× *g*. The precipitated pellets containing IgY were diluted in 10 mL of PBS using the same method described above and then precipitated once again with 12% of PEG 8000. The finished pellets were filtered through a 0.45 um filter, dissolved in 2.0 mL of PBS, and kept at −20 °C. SDS–PAGE and Coomassie blue staining were utilized to assess the purity of the lgY antibody.

IgY from egg yolk samples was investigated by using a protein profile of sodium dodecyl sulfate–polyacrylamide gel electrophoresis (SDS–PAGE) analysis. The SDS–PAGEs were run under both reducing and non-reducing conditions. SDS–PAGE was carried out using 12% polyacrylamide gels. The protein concentration was initially diluted to achieve a total protein content of 3 μg. Then, the sample was mixed with the Laemmli (with in/out 2—mercaptoethanol) loading buffer (2:1, *v*:*v*). The volume of the sample loaded was 10 μL/ lane, and 5 μL of IRIS11-stained protein ladder (Bio-Helix, Taiwan), which was utilized to compare molecular weights. SDS–PAGEs were performed using a Mini-PROTEAN^®^ Tetra System (Bio-Rad) in a tris/glycine/SDS running buffer for 60 min at a voltage of 120 V. The proteins were stained overnight with Coomassie Brilliant Blue G-250, then distained at room temperature. All gel images were analyzed using a ChemiDoc MP (Bio-Rad) [35,36].

### 3.5. Indirect Competitive ELISA Development

The 96-well immunoassay plate (Maxibinding; SPL) was coated with a coating antigen (CPFX-OVA) at 10 μg/mL overnight at 4 °C with 100 μL/well. The plates were washed four times with 0.05% PBST and blocked with 200 μL/well of 1% gelatin for 1 h. In another plate, the standards (ciprofloxacin, norfloxacin, and enrofloxacin) prepared at different levels in 1% skim milk PBST were added to wells (100 μL/well); then the antibody dilution 1:1000 was added (100 μL/well) and incubated for 1 h. The solution was discarded after blocking, then the mixed was transferred mix (antibody and standards) into the plate (100 μL/well) and incubated again for 1 h. Plates were washed fourice before adding 100 μL/well of goat anti-chicken IgY-HRP diluted (1:10,000) in PBST and incubated for 1 h. It was washed fourice again, and 100 μL/well of freshly prepared OPD solution was added and incubated for a final 30 min before the reaction was stopped by adding 2 N sulfuric acid (50 μL/well). All incubations were carried out in dark conditions. Finally, absorbance was read at 492 nm with the aid of a microplate reader [24,37].

### 3.6. Specificity Determination

Specificity, the capacity of the IgY polyclonal antibody (pAb) to recognize other structurally related compounds was expressed in a percentage and calculated using the given equations:

(1) $$\mathrm{CR}\%=\frac{{\mathrm{IC}}_{50}\;\mathrm{value}\;\mathrm{of}\;\mathrm{hapten}}{{\mathrm{IC}}_{50}\;\mathrm{value}\;\mathrm{of}\;\mathrm{copetiter}}\;\times \;100$$

### 3.7. Preparation of Chicken and Pork Meat Samples Extract

In the preparation process, the meat of chicken and pork were minced with fat and the skin separated. Three grams of the muscle sample was added into a 50 mL centrifuge tube along with 200 µL 0.1 M EDTA and 10 mL 70% *v*/*v* methanol. The mixture was vortexed for 30 s, shaken for 15 min, and centrifuged at 5000 rpm for 5 min. It was then diluted with 500 μL of extract with 2 mL of ultrapure water type I (final concentration of methanol is 14% *v*/*v*). Finally, it was then filtered by passing it through a 0.45 μm membrane filter [38].

### 3.8. Statistical Analysis and Curve Fitting

Analysis using statistics and curve-fitting the immunoassay data used a four-parameter logistic equation. Calculations were performed using Prism Pad 9 software (Prism Pad 9, San Diego, CA, USA).

## 4. Conclusions

The IgY antibody obtained from egg yolk immunized by mixed hapten is specifically effective on CPFX, NFX, and especially ENFX. The methanol concentration for extraction can used for analyzing fluoroquinolone residue at concentrations below 25% in the extract solution. The icELISA developed that could be detected during range IC_10_ (LOD)–IC_90_ were 0.37 μg/mL and 7.29 ng/mL, respectively. This demonstrates that IgY could be a potential option for detecting antibiotic residues in food samples and can be used for monitoring a large volume of samples and meeting the goal of food safety criteria.

## Figures and Tables

**Table 1:** Hapten density of immunogens and coating antigen (ciprofloxacin and norfloxacin conjugated).

No.		Sample ^1^	Hapten Density (Number of Amines/Protein)
1	Immunogens	BSA-Ciprofloxacin	0.0030 ± 0.0016
2		BSA-Norfloxacin	0.0107 ± 0.0030
3	Coating antigen	OVA-Ciprofloxacin	0.0021 ± 0.0006
4		OVA-Norfloxacin	0.0226 ± 0.0043

^1^ Hapten concentration of 890 μg/mL in 0.1 M sodium bicarbonate, pH 8.0, was prepared before reacting with 0.01% TNBS, incubating, and measuring at 420 nm with a spectrophotometer.

**Table 2:** The IC_50_ and percentage of cross-reactivities (%CR) of CPFX to other FQs in this icELISA study.

Compounds	Structures	Coating Antigen	IC_50_ (μg/mL)	Cross-Reactivity (%)
Ciprofloxacin	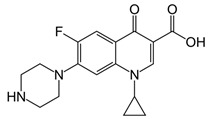	CPFX-OVA	0.80	100.00
		NFX-OVA	3.79	63.39
Norfloxacin	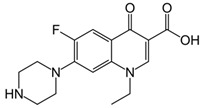	CPFX-OVA	0.63	126.98
		NFX-OVA	2.63	100.00
Enrofloxacin	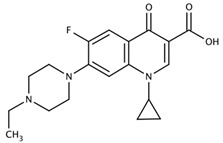	CPFX-OVA	0.05	1531.34
		NFX-OVA	2.66	98.87
Ofloxacin *		CPFX-OVA	>20.00	-
Levofloxacin *		CPFX-OVA	>20.00	-

* Exclusively coated CPFX-OVA was evaluated in this experiment.

**Table 3:** Percentage of recovery (%R) of the different concentrations of enrofloxacin evaluated in buffer and in matrices, mean recoveries and the coefficient of variation (CV) in meat-extracted matrices.

Analyte	Spiked Conc. (μg/mL)	Matrix (*n* = 2)					
		Chicken		Pork		Blank *	
		(%R)	(%CV)	(%R)	(%CV)	(%R)	(%CV)
Enrofloxacin	1.00	111.81–112.71	0.57	109.90–117.22	4.55	103.34–119.59	10.31
	0.10	96.69–103.62	4.89	98.45–103.00	3.19	99.22–103.64	3.08
	0.01	86.65–95.01	6.51	84.24–87.90	3.00	83.20–87.27	3.38

* Blank = the solution of 500 μL 70% *v*/*v* methanol was diluted in 2 mL ultrapure water type I.

**Table 4:** Comparison of various ELISA approaches for enrofloxacin determination.

Method	Immunogen	Antibody	IC_50_	Author (Year)
Immunochromatographic assays	ENFX-1-BSA	mAb (mouse)	0.03 ng/mL	Xianlu Lei et al. (2022) [23]
Indirect competitive enzyme-linked immunosorbent assay (icELISA)	ENFX-BSA	mAb (mouse)	0.15 ng/mL	Bitange Nipa Tochi et al. (2016) [24]
Indirect competitive fluorescence-linked immunosorbent assay (cFLISA)	ENFX-BSA	mAb	8.30 ng/mL	Junxia Chen et al. (2009) [25]
Chemiluminescent enzyme immunoassays (CLEIAs)	-	Aptamer (in vitro)	24.27 ng/mL	Hengjia Ni et al. (2014) [26]
Enzyme-linked immunosorbent assay (ELISA)	ENFX-BSA	pAb (rabbit)	70.00 ng/mL	Zhanhui Wang et al. (2014) [27]
Time-resolved fluoro-immunoassay (TRFIA)	ENFX-BSA	mAb (mouse)	1.83 μg/L	Zhen Zhang et al. (2013) [28]
Indirect competitive enzyme-linked immunosorbent assay (icELISA)	CPFX-BSA + NFX-BSA	pAb (chicken)	0.05 μg/L	This work
Ultraperformance liquid chromatography–tandem mass spectrometry (UPLC/MS/MS method)	-	-	0.05 μg/kg (LOD)	Bing Shao et al. (2007) [8]
High performance liquid chromatography with diode-array detection (HPLC-DAD)	-	-	0.096 μg/mL (LOD)	Elaine de Oliveira Araujo et al. (2020) [29]
Reversed phase–high performance liquid chromatography (RP-HPLC)	-	-	1.00 μg/mL (LOD)	Kandarp M. Patel et al. (2018) [30]

## Data Availability

Not applicable.
